# What good is weed diversity?

**DOI:** 10.1111/wre.12310

**Published:** 2018-05-23

**Authors:** J Storkey, P Neve

**Affiliations:** ^1^ Rothamsted Research Harpenden Hertfordshire UK; ^2^ IA USA

**Keywords:** niche differentiation, herbicide resistance, sustainable intensification, Broadbalk experiment, species richness

## Abstract

Should the declining diversity of weed communities in conventionally managed arable fields be regarded as a problem? The answer to this question has tended to divide researchers into those whose primary focus is on conserving farmland biodiversity and those whose goals are dictated by weed control and maximising yield. Here, we argue that, regardless of how weeds are perceived, there are common ecological principles that should underpin any approach to managing weed communities, and, based on these principles, increasing in‐field weed diversity could be advantageous agronomically as well as environmentally. We hypothesise that a more diverse weed community will be less competitive, less prone to dominance by highly adapted, herbicide‐resistant species and that the diversity of the weed seedbank will be indicative of the overall sustainability of the cropping system. Common to these hypotheses is the idea that the intensification of agriculture has been accompanied by a homogenisation of cropping systems and landscapes, accounting for both declines in weed diversity and the reduced resilience of cropping systems (including the build‐up of herbicide resistance). As such, weed communities represent a useful indicator of the success of rediversifying systems at multiple scales, which will be a central component of making agriculture and weed control more sustainable.

## Introduction

The number of weed species that are typically found in conventionally managed crop fields is now a fraction of the levels recorded in the 1950‐1970s, owing to increased fertiliser and herbicide use, simpler rotations and loss of field boundaries and semi‐natural features in the landscape (Andreasen *et al*., 1996; Fried *et al*., 2009; Meyer *et al*., 2013). For some, this loss of in‐field biodiversity is a concern, reflecting an erosion of the natural capital and ecosystem services on which sustainable production is founded. However, for others, it is seen as a measure of successful weed control and the concept of conserving weeds within cropped fields is, at best, incomprehensible and, at worst, an insult to the efforts of weed scientists over the past half‐century to reduce the serious yield losses inflicted by weeds (Oerke, 2006). This divergence in peoples’ perception of weeds represents a philosophical fault line running through the weed science community and often reflects differences in scientific background (ecology vs. agronomy). Here, we argue that, rather than perpetuating this dichotomy, we should recognise that the objectives of maintaining arable biodiversity and preventing cropping systems becoming dominated by a few highly competitive, herbicide‐resistant weeds both rely on a common set of ecological and management principles that should unite our research.

Two important points of clarification are needed to frame our argument. Firstly, the focus here is on weed species richness and evenness, not density or total biomass. There may be a benefit to increasing the total amount of plant resources for higher trophic groups on farmland (including seeds for farmland birds and pollen and nectar for pollinators and biocontrol agents), but the argument that weed biomass should be managed in crops specifically to provide these ‘ecosystem services’ is not our primary focus. Secondly, our emphasis is on the common weed flora rather than those rare and threatened arable specialists that have suffered the steepest declines in regional populations over recent decades and require specific conservation measures (Storkey *et al*., 2012). Our argument for increasing weed diversity is primarily agronomic and two pronged. Firstly, we contend that a more diverse weed community will be less competitive in any given crop and, secondly, that weed diversity is indicative of the wider sustainability of the whole cropping system.

## A more diverse weed community is less competitive

Ecological niche theory argues that phenotypic differentiation between species results in contrasting ability to capture the resources required for growth and that co‐existence is supported by spatial and temporal heterogeneity in resource availability (Silvertown *et al*., 1999; Chesson, 2000). While selective herbicides have been a major driver of recent declines in weed diversity, the homogenisation of habitats through the use of inorganic fertilisers and simplified crop rotations has also selected for fewer dominant species with similar resource requirements to the crop (Borgy *et al*., 2012). Although persistent seedbanks will continue to buffer the negative impacts of management, it is likely that the remaining diversity in conventionally managed crops is now driven to a much larger extent than in the past by repeated recolonisation from field edges or neighbouring ruderal habitats. This may explain the large numbers of ‘chance’ records observed in modern weed surveys (Baessler & Klotz, 2006) and why diversity is higher in complex landscapes with smaller fields (Gabriel *et al*., 2005; Gaba *et al*., 2010).

**Figure 1:**
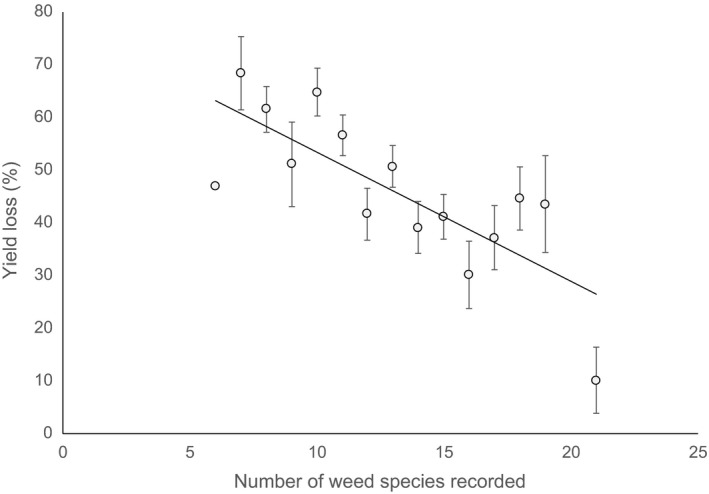
Relationship between weed species richness and crop yield loss on the Broadbalk winter wheat experiment (begun in 1843). Weed species richness is assessed on herbicide‐free plots annually, and weed diversity varies in response to contrasting fertiliser treatments. Winter wheat yield loss from weed competition can be calculated as a percentage of the equivalent plots with the same fertiliser treatments but where weeds are controlled with herbicides. Data are presented for 19 years collected between 1991 and 2014 (the plots were fallowed in some years during this period) and sorted by weed species richness. For each level of weed species richness, the average yield loss is presented with error bars indicated the standard error of the mean (*r* ^2^ = 0.59, *P *<* *0.001). Plots with no nitrogen but with added phosphorus and potassium are excluded from the analysis as the abundance of leguminous weeds leads to facilitation and greater yield in the weedy plots compared to the weed‐free plots.

Smith *et al*. (2010) explored the hypothesis that one consequence of a reducing niche breadth for weeds is that interspecific competition with the crop intensifies as the diversity of available resource pools decreases – the so‐called Resource Pool Diversity Hypothesis (RPDH). In unmanaged systems, the supply and imbalance of limiting resources have also been postulated as the direct proximate cause of variation in species richness (Cardinale *et al*., 2009). Where increased weed diversity is an *emergent property of spatio‐temporal heterogeneity in resource supply*, therefore, we would expect decreased crop competition. There is now some support for this hypothesis in the literature (Cierjacks *et al*., 2016), in contrast to experiments that use artificially assembled weed communities which can be confounded by the ‘sampling effect’ (Pollnac *et al*., 2009). Here, we provide further support for the RPDH using data from the Broadbalk long‐term experiment at Rothamsted (Fig. 1). The Broadbalk experiment includes herbicide‐free plots with contrasting fertiliser treatments and naturally assembled weed communities that we have compared with equivalent weed‐free plots to calculate yield loss. A classic ‘hump‐backed’ relationship of weed species richness is observed along the fertility gradient with the highest species richness observed on intermediate fertility plots where soil resources are most evenly balanced (Moss *et al*., 2004). When the data were grouped on the basis of weed species richness, a strong negative relationship with crop yield loss was observed.

The concept of habitat heterogeneity can be extended beyond fine‐scale spatial patchiness of soil resources to capture a range of other management interventions that act as filters on the local weed species pool (Booth & Swanton, 2002); examples include crop sowing date and intensity of cultivation. In functional terms, a greater diversity of crop types, nutrient inputs and cultivation practices will lead to a greater breadth of weed ‘response’ traits, consequently reducing the dominance of competitive ‘effect traits’ that impact crop yield (Navas, 2012). Robust evidence supporting this hypothesis of weed competition based on functional traits is currently lacking in the literature. In providing this evidence, long‐term cropping experiments that result in naturally assembled gradients of weed functional diversity are preferable to short‐term studies on artificially selected weed mixtures. Additional, trait‐based, analysis of existing experiments such as Broadbalk and the establishment of new long‐term cropping system experiments that focus on weed diversity and crop yield loss should, therefore, be a key research aim in the future.

## A more diverse weed community is an indicator of agronomic and environmental sustainability

The simplification of cropping systems and increased inputs of agro‐chemicals have led to the dominance of a few competitive, highly adapted and widely distributed weed species. This is exemplified by *Alopecurus myosuroides* Huds. in north‐west Europe (Délye *et al*., 2010), *Amaranthus palmeri* S. Watson in southern and central United States (Ward *et al*., 2013) and *Lolium rigidum* Gaudin in Australia (Owen *et al*., 2014). As populations of these species increase, growers become more reliant on fewer herbicides, selection pressure increases and dominant weeds quickly adapt to new management including becoming resistant to herbicides (Neve *et al*., 2014). Many of the world's cropping systems are now afflicted by herbicide resistance, such that as weed diversity has declined, in many cases weed biomass has not; this challenges the ongoing sustainability of the whole system. Herbicide resistance is just one of multiple factors currently threatening the agronomic and environmental sustainability of modern agriculture. Other pressures on the system include declining soil health, pollution of water courses, greenhouse gas emissions and declining functional biodiversity (including pollinator populations). The loss of agro‐ecosystem diversity has contributed to these problems; the cultivation of a narrow range of functionally similar crops on large contiguous areas is predicated on highly mechanised systems with high inputs of inorganic fertiliser and pesticides, leading to open, leaky systems with minimal organic inputs to the soil.

In response to these stresses, there is a call for the ‘sustainable intensification’ (SI) of agriculture that increases food production without further adverse environmental impacts. This is a multifaceted and complex challenge that will require trading off multiple criteria using a range of metrics (Garnett *et al*., 2013). As well as being a direct threat to agronomic sustainability, we hypothesise that recent declines in weed species richness are correlated with the wider loss of cropping system resilience and that weed communities may represent a useful proxy for agronomic and environmental sustainability at the field, farm and landscape scale. There is potential, therefore, to develop an *indicator of sustainable intensification* built on the taxonomic or functional diversity of the weed seedbank that represents a legacy of previous management across the whole cropping system. Our hypothesis is that fields with low seedbank diversity have a less sustainable cropping system, both agronomically and environmentally, than a field with a more diverse weed community. Challenging this thesis will require a research effort to establish relationships between weed diversity and other metrics of SI, including soil health and functional biodiversity and the development of protocols that identify the appropriate measure of weed diversity. Because the emerged weed community in any given year is determined by the management specific to the crop being grown, as well as stochastic processes, sporadic assessments of the above‐ground flora are not appropriate for this purpose. Rather, continuous assessments over the whole cropping system or sampling of the seedbank are required to capture the response of the weed flora to the range of management practices applied across the whole cropping system.

In conclusion, as weed biologists working at a single institution but whose research focusses on environmental and production endpoints respectively, we are convinced that the loss of weed diversity and the escalation of resistance to herbicides are mediated by an identical underlying cause: the simplification of agroecosystems and their associated weed management strategies. Given this, we propose that the goals of designing weed management systems that maximise production and maintain ecosystem functioning are entirely compatible and mutually reinforcing. We would, therefore, echo the call made by previous authors (Fernandez‐Quintanilla *et al*., 2008; Jordan & Davis, 2015) for weed scientists to integrate their work within the transdisciplinary framework that is required to meet the challenge of sustainable intensification and the transformation of cropping systems. In so doing, we would move weed science from being a parochial discipline towards an integral part of a broader research effort focussed on transforming the current, flawed paradigm of modern intensive agriculture.
